# Iodine, a Remedy in Rattlesnake Bites

**Published:** 1856-02

**Authors:** Benj. Woodward

**Affiliations:** Sharon, Ill.


*Iodine, a Remedy in Rattlesnake Bite.*

CASE II.—On the evening of the last 4th of July, I was called to see a little Dutch girl, 12 years old, who had been bitten early in the morning by a rattlesnake.

I found the wound on the inside of the right ancle, two marks of the fangs were distinctly visible, from which bloody serum was issuing. The whole limb and right side were enormously swelled and discolored. Tongue swelled, pulse 140 per minute, delirious, constant vomiting of green slime or mucous, abdomen tympanitic.

Here was a case to try Dr. Brainard's remedy. I should have mentioned before, that in many places the skin of the leg was full of cracks, from which issued yellow serum. Gave morphine in $\frac{1}{2}$ gr. doses every half hour, to allay the vomiting and to quiet the brain.

I scarified not only the limb, but almost every part of the right side, and then rubed in strong tinct. of iodine, and as soon as the vomiting was allayed, gave a tea spoon-full every hour of solution of iodide potassa, 20 grs. to the ounce of water, moved the bowels with enema of epsom salts, repeated till effect was produced.

Saw her again on the morning of the 5th, she was rational, and complained of the pain in her leg and thigh. Scarified again, and washed with the tinct. iodine. Ordered elm mucilage, and the iodide potssa in tea spoon-full doses every four hours.

On the 6th, found all symptoms relieved, discontinued the internal use of the iodine as it affected the bowels materially. Gave pulv. doveri 10 grs., quinine 4 grs., every six hours till four doses had been taken.

7th, found her comfortable, except some lameness of wounded limb. Ordered simple soothing applications and dismissed the case.

I would not claim for myself any credit in this case, but would give it to the man who so successfully experimented with the iodine in animal poisoning. The above, from the length of time that had elapsed before remedies were used, and the severity of the symptoms, seemed a desperate case, and yet the iodine triumphed over the poison.

Why may not iodine prove a remedy in that most terrible of all diseases, canine madness? That is caused by an animal poison most certainly. True its effects are seen mostly in the nervious system, and even if the remedy will not control the disease after it is developed—may it not have the effect of eliminating the poison before actual madness takes place.

So impressed am I with this idea, that I propose to institute a series of experiments with the canine virus as soon as I can procure it. To this end, I will thank any member of the profession to send me by mail, at my expense, any specimens of the virus he can procure, either from the recently dead, or living animal. It may be procured and preserved by collecting the saliva on pieces of quill, drying them, and putting them into dry, well corked vials. Should any gentleman be so kind as to send me any, I will send him the results of the experiments. I have not been able to procure any myself, since the idea first suggested itself to my mind.

Benj. Woodward, M. D.

*Sharon, Ill., Nov.* 1, 1855.

